# The Use of Artificial Intelligence in Idiopathic Clubfoot: A Scoping Review

**DOI:** 10.5435/JAAOSGlobal-D-25-00376

**Published:** 2026-10-14

**Authors:** Rony Imad El Hachem, Jean-Paul Rizk, Imad Nahle

**Affiliations:** From the Department of Orthopedic Surgery, American University of Beirut Medical Center, Beirut, Lebanon.

## Abstract

**Background:**

The past decade has been marked by the progressive integration of artificial intelligence (AI), especially in the form of machine learning and deep learning. Pediatric orthopaedics remains an underexplored area in AI, especially in the context of congenital deformities such as clubfoot.

**Objective:**

A scoping review would help organize the various uses of AI in this domain by identifying studies and categorizing them based on the technique used and their applications, exposing gaps, and informing future studies to support AI integration in pediatric care.

**Methods:**

A Preferred Reporting Items for Systematic Reviews and Meta-Analyses–based analysis was conducted, and several databases were used to scope for articles (MEDLINE, PubMed, Scopus, Google Scholar) using a set of keywords and mapped-out terms.

**Results:**

A total of 16 studies met all eligibility criteria and were included in this review. These studies covered the use of AI, machine learning, or computational modeling over a broad range of objectives such as diagnosis, treatment, gait analysis, and prognostic modeling.

**Conclusion:**

AI is moving from promise to practice in clubfoot diagnosis, decision making, and even relapse detection. Indeed, across 16 studies, AI applied as different forms, such as deep learning classifiers and finite element models, shows potential to offer solutions to long-standing clinical challenges such as subjective assessments and variability in treatment.

## Introduction

The past decade has been marked by the progressive integration of artificial intelligence (AI), especially in the form of machine learning (ML) and deep learning (DL).^1^ AI allows the processing of large volumes of data and suggestion of clinical patterns that aid in both diagnostic and prognostic decisions.^2^ Several fields have been adopting AI use, such as radiographic interpretation, skin lesion classification, and detection of orthopaedic pathologies; DL algorithms match the expert performance of clinicians in them.^1^ Thus, AI has been implicated in clinical settings to assist with risk stratification, outcome prediction, and treatment optimization.

Nonetheless, pediatric orthopaedics remains an underexplored area by AI, especially in the context of congenital deformities such as clubfoot, that is, congenital talipes equinovarus. Clubfoot, defined as a three-dimensional deformity, is a common musculoskeletal birth defect affecting approximately one in every 1000 live births.^3,4^ Epidemiologically, the condition is more common in male individuals, with a ratio of 2:1, and is mostly idiopathic, affecting otherwise healthy children, although other forms are present.

Several approaches to treat clubfoot have been proposed. The Ponseti method has remained the mainstream treatment over the past decades. It is divided into three phases: manipulation and serial casting, percutaneous Achilles tenotomy in around 80% to 90% of the cases, and a prolonged period of bracing treatment. If applied correctly with adequate patient and family compliance, the Ponseti method shows a success rate above 90%.^4^ Nonetheless, the relapse rate remains noticeable and can reach 20% to 30% by age four, even in experienced centers.^5^

This explains why early identification of high-risk children for diagnosis and recurrence remains an important need. Classifications systems such as the Pirani or Dimiglio have been developed, which gives some guidance in assessing severity and monitoring treatment progress.^6^ Nonetheless, their predictive value is limited, and variability in clinician experience and interpretation of physical findings adds to the uncertainty. Other modalities such as MRI or radiographs, ultrasonography, and instrumented gait-laboratory analysis have been used to identify parameters that help predict treatment outcomes.^7^

Therefore, AI and the different models hold potential in treating this condition. Through applications such as image analysis, motion tracking, and brace wear-time prediction, AI can help in early diagnosis and forecasting relapses. This potential remains unexplored, and the literature is still fragmented and lacks systematic analysis. Early studies focus on different approaches, but no comprehensive review to map the uses and applications has been written. Following this rationale, a scoping review would help organize the various uses of AI in this domain by identifying studies and categorizing them based on the technique used and their applications, exposing gaps, and informing future studies to support AI integration in pediatric care.

## Methods

### Protocol and Reporting Guidelines

This scoping review was conducted following the Preferred Reporting Items for Systematic Reviews and Meta-Analyses extension for Scoping Reviews guidelines. The review protocol was developed collaboratively by the authors and adhered to best practices for scoping methodology, ensuring transparency and reproducibility throughout the review process. Auto-alerts were created to ensure that no issues were missed by the time of submission.

### Eligibility Criteria

We applied the following inclusion and exclusion criteria to map the applications of AI in diagnosis, treatment, and recurrence of idiopathic clubfoot.

#### Inclusion Criteria

- Studies published between 2015 and 2025.
- Peer-reviewed articles.
- Studies involving human pediatric patients with idiopathic clubfoot.
- Studies reporting on the use of AI methods (eg, ML, DL, neural networks, and predictive algorithms).

#### Exclusion Criteria

- Studies focusing on syndromic, neurogenic, or secondary clubfoot etiologies.
- Animal studies, editorials, letters, and non–peer-reviewed content.

### Information Sources

A comprehensive search was conducted in MEDLINE, Scopus, PubMed, and Google Scholar.

### Search Strategy

The search conducted on MEDLINE will be shown here. The other searches will be made available in the index for replicability.

#### OVID

Ovid MEDLINE(R) Epub Ahead of Print and In-Process, In-Data-Review & Other Non-Indexed Citations and Daily < June 16, 2025>.

1. Clubfoot/or equinus deformity/or talipes cavus/4722
2. (clubfoot or clubfeet or equinovarus or “equino varus” or Congenital Talipes Equinovarus (CTEV) or “congenital talipes” or “congenital foot deform*” or “pie torcido*”).mp.6073
3. 1 or 2 6584
4. exp Artificial Intelligence/239674
5. (“neural network*” or “computer vision” or “predictive algorithm*” or “decision support system*” or “AI model*” or “ML model*” or “DL model*” or “intelligent system*” or “smart algorithm*” or “large language model*” or “cognitive technology” or “cognitive computing” or “cognitive robotics” or ChatGPT or genAI or “gen-AI” or “creative AI” or “generative AI”).mp.197638
6. ((artificial or machine or computer* or deep or predictive or automated or neural or ensemble or federated or ambient) adj3 (intelligence or learning or reasoning or model* or network* or detection or diagnosis or prediction or acquisition or representation)).mp.522920
7. 4 or 5 or 6 600326
8. 3 and 7 15
9. limit 8 to yr = “2015 -Current” 8

https://ovidsp.ovid.com/ovidweb.cgi?T=JS&NEWS=N&PAGE=main&SHAREDSEARCHID=6fe4LvY7WjKD4IUg1bibQsLBr41b7DiNMWm53Url35iKmOGU9BF8IpxFABvY9HUO5.

### Study Selection

All search results were imported into a shared screening document. Duplicate records were removed. Two reviewers independently screened titles and abstracts for eligibility. Full-text articles were then retrieved and assessed against the inclusion/exclusion criteria. Disagreements were resolved by consensus.

## Results

### Study Selection

The initial database search yielded a total of 485 records: PubMed (n = 267), Google Scholar (n = 200), Scopus (n = 39), and MEDLINE (n = 10). After removal of 32 duplicate entries, 452 unique studies were screened by title and abstract. At this stage, records were excluded based on lack of relevance to idiopathic clubfoot or AI (as well as computational methods), or on focus on syndromic conditions, as well as unrelated congenital syndromes.

After this title and abstract screening phase, 25 studies seemed potentially eligible. After full-text review, seven studies were excluded because of not meeting the inclusion criteria as listed.

A total of 16 studies met all eligibility criteria and were included in this review. These studies covered the use of AI, ML, or computational modeling over a broad range of objectives such as diagnosis, treatment, gait analysis, and prognostic modeling.

### Characteristics of Included Studies

The 16 included studies, published between 2017 and 2025, show variability in their design and objectives, showcasing the myriad applications of AI in managing clubfoot. Some of the study designs seen are retrospective observational studies, prospective analyses, case reports, and methodological development studies. Not only did the designs vary but so did the different sample sizes, ranging from single-patient studies to hundreds of patients.

#### Diagnostic and Classification Artificial Intelligence

##### Plantar Pressure–Based Classification

Three studies used plantar pressure to discriminate foot types and conditions, which included talipes equinovarus as well:

- A Convolutional Neural Network (CNN) optical sensor plantar pressure was efficient in classifying the conditions under a supervised setup.^8^
- A residual network reported a 95% class accuracy across nine foot types.^9^
- Synthetic plantar pressure data were generated by a Gaussian mixture model and generative adversarial network (GAN) and improved accuracy if used with ML classifiers.^10^

##### Movement and Gait-Based Classification

- A retrospective data set of 1211 patients and 61 control subjects used an InterllEVENT, which is an Long Short-Term Memory (LSTM)-based algorithm to detect initial contact and foot-off with a rate of 99% and 95%, respectively, in different conditions, clubfoot included.^11^
- A study using kinematics was able to detect relapse versus nonrelapses and showed that the usage of dynamic activities in combination improves sensitivity.^12^

##### Radiographic-Based Measurement

- A convolutional neural network (CNN) model was able to calculate angle measurements on pediatric foot radiographs.^13^

##### Biomechanical Inferences Based on Kinematics

- In a pediatric cohort of 13 patients with bilateral CTEV and 17 typically developing children, random forest and deep neural networks predicted joint moments from kinematics with correlations of approximately 0.74 to 0.76 and Normalized Root Mean Square Error (nRMSE) of approximately 23%, noting the potential of their application but limited accuracy.^14^

##### Explainable Artificial Intelligence for Foot Conditions

- An explainable ML pipeline (feature engineering + Support Vector Machine (SVM)/Random Forest (RF)/K-Nearest Neighbors (KNN)/Logistic Regression (LR), with Local Interpretable Model-Agnostic Explanations (LIME) achieved balanced accuracy and F1 ≈ 0.87 across six foot conditions.^15^

Thus, AI can use imaging, plantar pressure, and kinematics to classify conditions and detect events along with biomechanical estimations.

##### 3D Modeling and Orthosis Design

- A 3D assessment protocol was able to define a pipeline for clubfoot evaluation, setting standards for measurement foundations.^16^
- A 3D-based CT evaluation was able to quantify the bone-to-bone relationship in clubfoot, using 3D metrics.^17^
- A personalized treatment planning using a finite element case report was described and included end-on-elongation estimates (eg, Achilles 2.71 cm and anterior tibialis 1.69 cm) and von Mises stress (∼4.26 MPa) within elastic limits, with complete correction in 3 months.^18^
- Image reconstruction with an orthosis conceptual design was used to help in mapping clubfoot geometry during correction, showcasing the change in device design.^19^

#### Digital Health and Information

- AI-generated clubfoot information using CHATGPT-4.0, Meta AI, and Gemini showed a mean clarity of 3.4, an accuracy of 3.0, and a safety of 3.3 on a four-point scale, with only 30% being entirely safe for search, showing risks if unsupervised for patient use.^20^ Nonetheless, some models such as ChatGPT-4o relied more heavily on academic and scholarly information than other models such as Google.^21^
- Self-Monitoring Analysis and Reporting Technology (SMART) sensors used in foot abduction orthosis compliance in CTEV proved useful in monitoring adherence.^22^

#### Genomics and Bioinformatics Evidence

- Profiler, NetworkAnalyst, and WebGestalt were integrated in a bioinformatics analysis highlighting apoptosis, Extracellular Matrix (ECM), muscle contraction, morphogenesis, and immune pathways, with implicated Transcription Factors (TFs) (eg, FOXN3, GLI3, HOX, NCOR2) and genes (APAF1, BCL2, Caspase (gene family) (CASP) family, Methylenetetrahydrofolate Reductase (gene) (MTHFR), Tropomyosin (gene family) (TPM), proposing additional etiological mechanisms (eg, cellular/immune responses to stimuli) for clubfoot.^23^

A Preferred Reporting Items for Systematic Reviews and Meta-Analyses–based flow diagram illustrating study identification, screening, and inclusion is shown in Figure 1.

**Figure 1:**
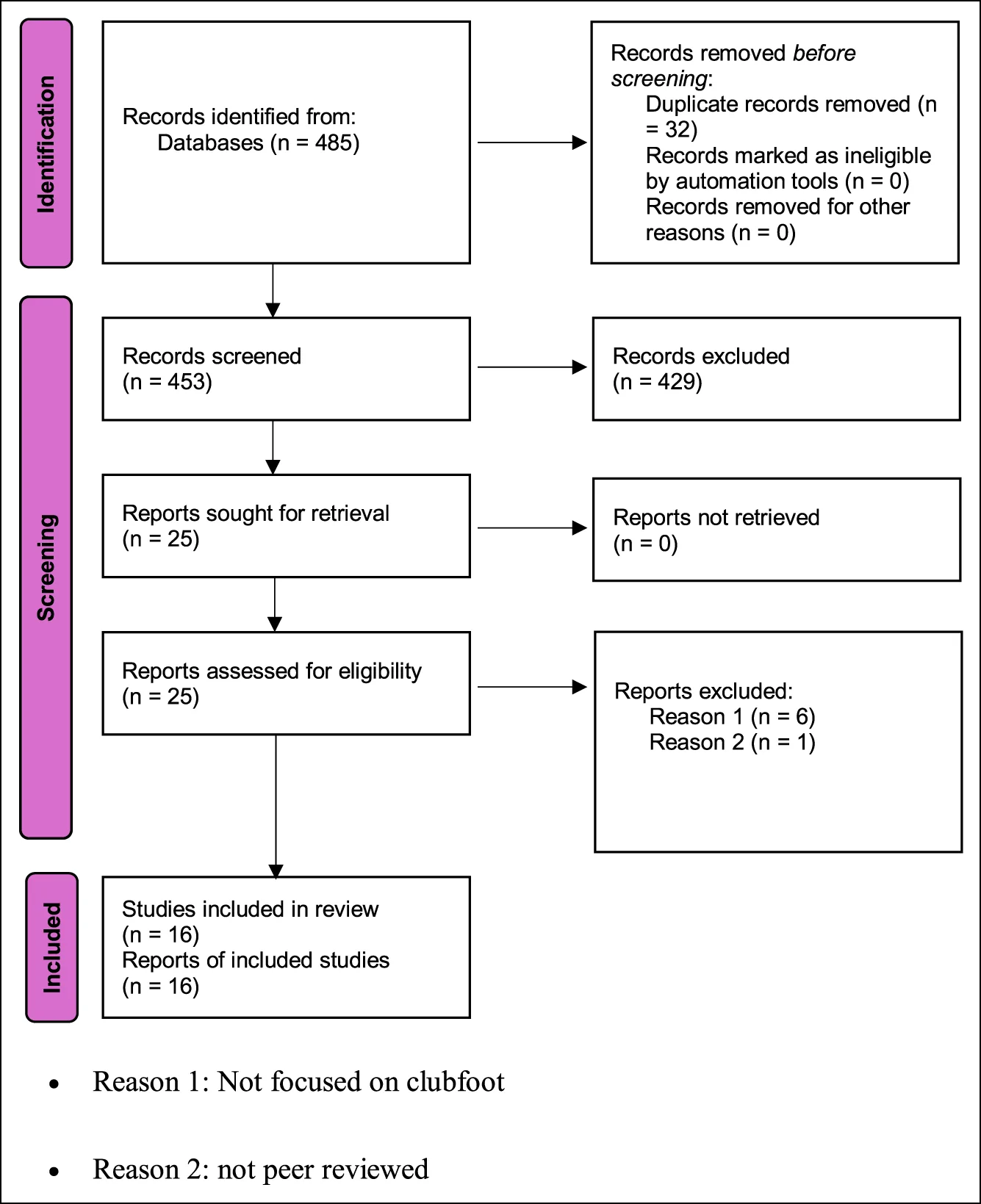
PRISMA flow diagram exhibiting the search strategy adopted across the databases. PRISMA = Preferred Reporting Items for Systematic Reviews and Meta-Analyses

## Analysis and Discussion

Across the 15 studies included, AI pivots around four axes in the care of clubfoot. AI plays a role in diagnostic and imaging analyses, and it also helps in determining prognosis and relapse. 3D modeling and simulation are also done, and it supports decision making.

### Diagnostic Analytics

DL pipelines based on plantar pressure or footprint images show potential in categorizing and discriminating between different foot conditions, including clubfoot, with minimal acquisition burden. For example, a residual network/conditional-GAN approach showed a 95% accuracy across the different categories of foot conditions: normal, planus, and talipes equinovarus. This engineering feat showcases the feasibility of pixel-level classification.^9^ In addition, the practicality of automated screening through CNN-based pressure sensor imaging has been shown.^8^ The accuracy can also be augmented by using generative strategies that synthesize plantar pressure data, such as Gaussian mixture model or 1D-GAN, to further tackle small data constraints.^10^ Imaging is advancing, and radiographic automation is also progressing. Indeed, a pediatric foot radiograph CNN was able to measure angles, showing the capacity of replacing or even standardizing the manual measurements, which is considered a source of variability.^13^

To extrapolate beyond imaging, clinical gait analysis through ML systems also showed promising accuracy metrics of 0.87 and offered interpretability that aligns with clinical reasoning.^15^

These tools are promising for longitudinal assessment, especially in contexts where subspecialty expertise might be scarce.

### Prognosis and Relapse Detection

Despite the fact that prognostic models remain scarce, they are clinically pivotal. A study shows that ML models trained on kinematics are able to estimate joint moments from gait without force plates. This is especially useful where kinematic capture is practical. Nonetheless, the models are still limited and need improvement before reaching clinical deployment.^15^ To go further, a developed model was able to use lower-limb kinematics, that is, hip, pelvis, knee, ankle, and foot, to show that explainable classifiers can distinguish relapsed children from nonrelapsed children. Sensitivity is also enhanced by combining activities such as toe walking, which allowed the model to flag children before the relapse was clinically recognized.^12^ These studies open a path toward earlier alerts that can be integrated with clinical judgment and decisions.

### D Modeling and Foundations for a Digital Twin

3D modeling is being developed to ameliorate objective assessment and planning. Reproducible pipelines for subsequent modeling and analysis are being established by 3D clubfoot assessment and shape capture.^16^ A patient-specific finite element modeling showed situation-based optimization of force distribution and tendon elongations to guide therapy. This feat acts as a step toward a digital twin concept in which corrections can be tested in silico before the intervention.^18^ Moreover, 3D evaluation methods that quantify bone-to-bone relations could also help in harmonization with the finite element modeling–based analysis to better predict future outcomes.^17^

### Decision Support and Workflow Integration

Prototypes of decision support pipelines show diversity, including automated measurements, orthosis conceptualization, and sensor-based monitoring. Conceptual orthosis design based on reconstruction and computational assessment might help in developing customized bracing treatment.^19^ To go further, SMART technologies in orthopaedics open the door for instrumented implants, which offer real-time monitoring and gait-linked feedback. These features could prove helpful in patient adherence and even remote follow-ups.^22^

### Information Quality and Safety

Since the public use of chatbots has soared, notable concerns regarding Frequently Asked Questions (FAQs) have been seen during the evaluation of generative models. Indeed, up to 25% of the outputs were inaccurate or limited, and only 30% were deemed safe. Therefore, misinformation and patient safety are on the line, which are particularly salient in pediatric populations, where trust and ease of use are critical for uptake. However, it is important to note that some models such as ChatGPT-4o provided information as accurately as Google and even relied more on scholarly sources for clinically relevant questions.^21^ This strengthens the urge to update and support AI chatbots using clinicians' oversight.^20^

### Limitations

Data set scale and diversity remain constrained. Many of the models relied on single-center cohorts, making external validity uncertain. Even high-performing pipelines can degrade over acquisition setups.^11^ Moreover, validation standards are heterogeneous. Some reports used internal metrics such as accuracy and Intraclass Correlation Coefficient (ICC); nonetheless, external testing remains limited.^9^ Finally, etiologic and bioinformatics bridges to phenotypes remain underexplored. Network analysis of candidate genes and pathways helps generate hypotheses, but links to other factors, such as imaging and gait analysis, are not yet integrated into a multimodal prognostic model.^23^

### Practical Implications

Practically, progress is now based on building multicenter data sets using carefully synthesized augmentation to stabilize training. This approach is promising and could improve plantar pressure classification accuracy if added to limited corpora.^10^ In bedside practice, models that prioritize signaling clinicians about foot conditions and their classification could be used to expose clinically useful gait features and even highlight earlier relapses than a routine clinical follow-up.^12,15^ Moreover, noninvasive classifiers show high accuracy, making them a low-burden screening tool if calibrated and validated.^9^

## Conclusion

AI is moving from promise to practice in clubfoot diagnosis, decision making, and even relapse detection. Indeed, across 16 studies, AI applied as different forms, such as DL classifiers to finite element models, shows potential to offer solutions to long-standing clinical challenges such as subjective assessments and variability in treatment. Yet, despite the advances, the field remains in its early stages. If AI is to transition to the clinical context, future work must prioritize external validation and integration into care. Ultimately, AI holds considerable promise as a tool to augment, and not replace, clinical expertise. However, realizing this vision will require sustained collaboration, responsible development, and a steadfast commitment to clinical relevance.
